# Supplementary figures and images for: Empirical analysis of COVID-19 confirmed cases, hospitalizations, vaccination, and international travel across Belgian provinces in 2021
Source: PLoS One. 2025 May 23;20(5):e0322017. doi: 10.1371/journal.pone.0322017 (PMC12101632; doi:10.1371/journal.pone.0322017)

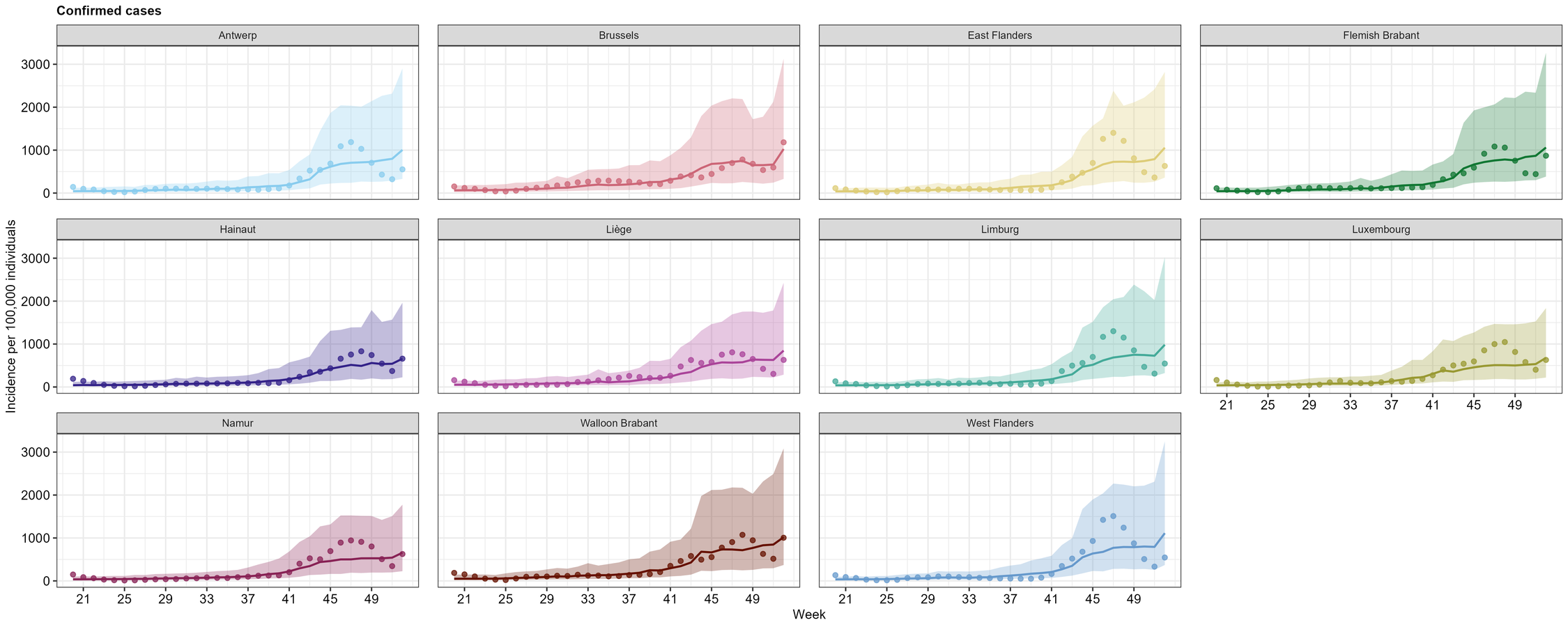

Supplement: S1 Figure — (TIF) [file pone.0322017.s003.tif]

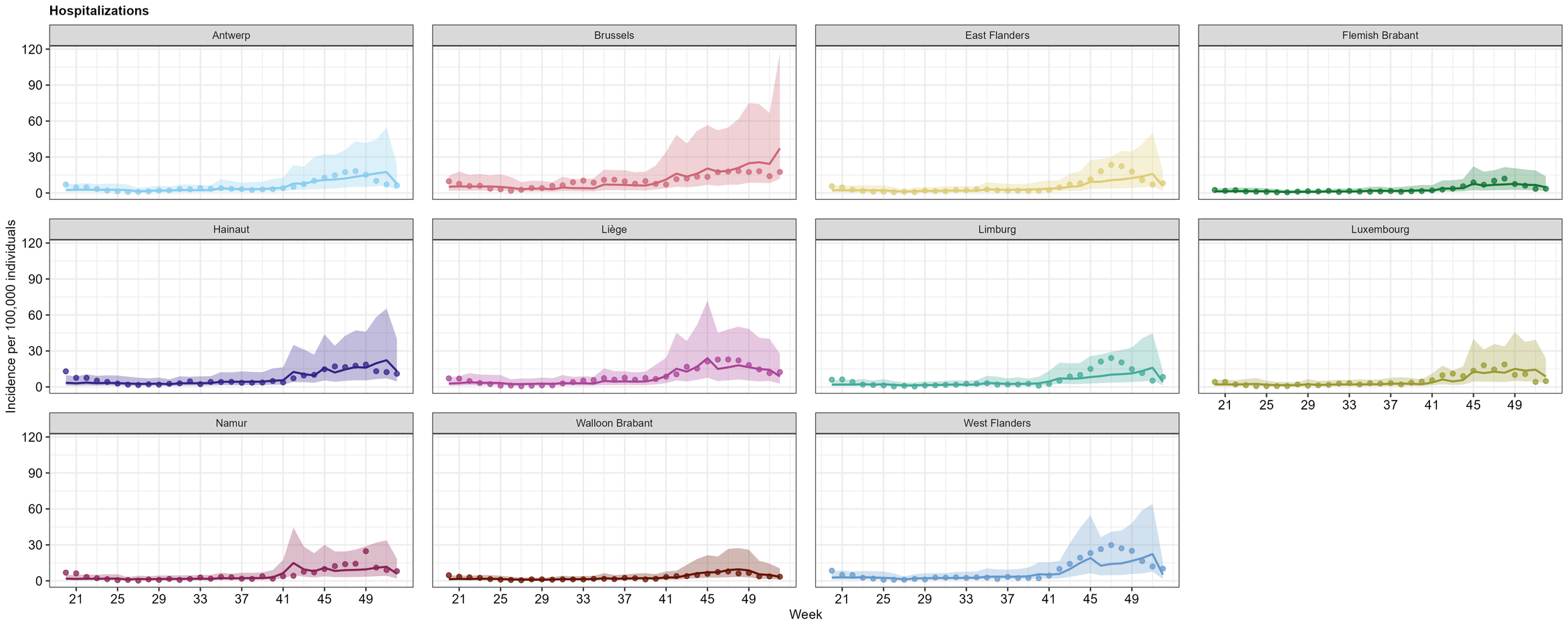

Supplement: S2 Figure — (TIF) [file pone.0322017.s004.tif]

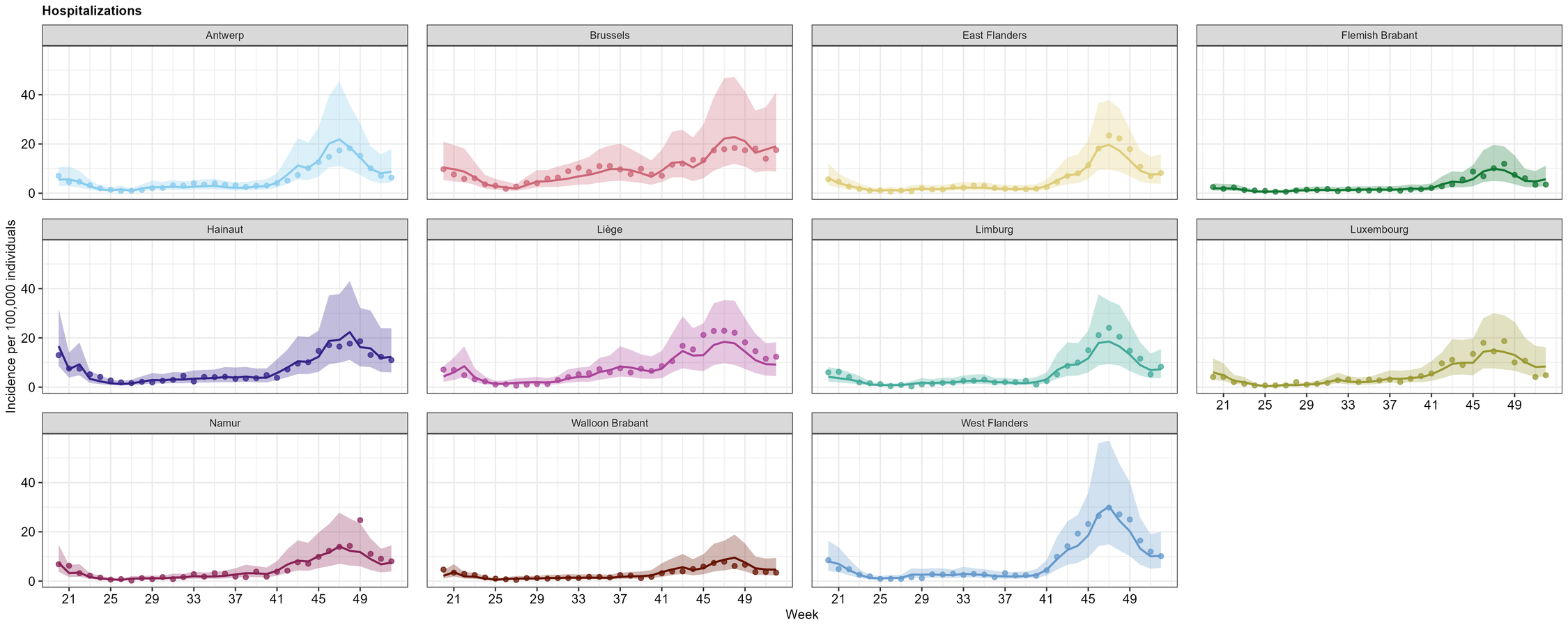

Supplement: S3 Figure — (TIF) [file pone.0322017.s005.tif]
