# Supplementary material for: Empirical analysis of COVID-19 confirmed cases, hospitalizations, vaccination, and international travel across Belgian provinces in 2021
Source: PLoS One. 2025 May 23;20(5):e0322017. doi: 10.1371/journal.pone.0322017 (PMC12101632; doi:10.1371/journal.pone.0322017)
Supplement: Attachment — Submitted filename: Response to reviewers.pdf [file pone.0322017.s006.pdf]

Empirical analysis of COVID-19 confirmed cases, hospitalizations, vaccination, and international travel across Belgian provinces in 2021

Yessika Adelwin Natalia[1], Geert Molenberghs[1,2], Thomas Neyens[1,2], Niel Hens[1,3], and Christel Faes[1]

[1] *I-BioStat, Data Science Institute, Hasselt University, Hasselt, Belgium*

[2] *I-Biostat, Leuven Biostatistics and Statistical Bioinformatics Centre, KU Leuven, Leuven, Belgium*

[3] *Centre for Health Economics Research and Modelling of Infectious Diseases (CHERMID), Vaccine and Infectious Disease Institute (VAXINFECTIO), University of Antwerp, Antwerp, Belgium*

Response to reviewers

First and foremost, we want to thank the reviewers for their critical assessment of our manuscript and their constructive comments. We have addressed all of them and modified the paper accordingly. For your convenience, the reviewers' comments have been listed in order below in italics, followed by our responses in regular typeface. To provide a concise response, we have grouped some comments related to a certain topic or part of the manuscript into one point for each reviewer.

Academic editor

Q AE.1 *Please ensure that your manuscript meets PLOS ONE's style requirements, including those for file naming. The PLOS ONE style templates can be found at https://journals.plos.org/plosone/s/file?id=wjVg/PLOSOne_formatting_sample_main_body.pdf and https://journals.plos.org/plosone/s/file?id=ba62/PLOSOne_formatting_sample_title_authors_affiliations.pdf.*

Reply: The manuscript is formatted using PLOS ONE LaTeX style formatting (Version 3.6 Aug 2022) found at `https://journals.plos.org/plosone/s/file?id=SyJ3/plos-latex-template.zip`. The author affiliations and file naming are adjusted to meet the journal's requirements.

Q AE.2 *We note that [Figure 1] in your submission contain [map/satellite] images which may be copyrighted. All PLOS content is published under the Creative Commons Attribution License (CC BY 4.0), which means that the manuscript, images, and Supporting Information files will be freely available online, and any third party is permitted to access, download, copy, distribute, and use these materials in any way, even commercially, with proper attribution. For these reasons, we cannot publish previously copyrighted maps or satellite images created using proprietary data, such as Google software (Google Maps, Street View, and Earth). For more information, see our copyright guidelines:* `http://journals.plos.org/plosone/s/licenses-and-copyright`.

Reply: These figures were made using map images from a public domain website located at `https://statbel.fgov.be/en/open-data`. These open data are free to use for commercial as well as non-commercial purposes. The licence can be found at: `https://statbel.fgov.be/sites/default/files/files/opendata/Licence%20open%20data_EN.pdf`, which states that we only need to refer to the source and date of publication. We developed all images in this study with these publicly available data. We uploaded the permissions as an "Other" file with our submission.

Reviewer 1

The paper explores the association between COVID-19 confirmed infections and hospitalizations on one hand and vaccination and travel on the other hand in provinces and regions of Belgium in a short and specific timeframe (April-July 2021). The aim of the paper is not very clear to me, which is partly due to the structure of the paper, which is more of a chronological description of an explorative modelling exercise. For example, in section statistical method: first... in regional level: then,... in provincial level the last sentence, linear regression section: first,..then,... Moreover, the writing is in some instances grammatically poor and statements incomplete or simply inaccurate.
This paper require substantial improvement to make it publishable. I would recommend the authors to restructure the paper and bring the association message forward based on the authors assessment of the best model and move the modeling exercise to the appendix.

Reply: Thank you very much for your time and effort in reviewing our work. We have thoroughly revised the manuscript based on these and the following comments.

Q 1.1 *It is insufficiently motivated why the very short time-frame was chosen. For example if travel is of interest, why did the authors stopped the analyses in the middle of the summer period, during which one anticipates many traveling. Would it not be interesting to continue the analysis until after the summer period, into September/October to see perhaps a reverse effect on infections/hospitalizations due to decreased traveling? Certainly the association with travel increase and simultaneous increase in incidence and hospitalization it is interesting to see what happens after the summer period.*

Reply: Thank you for this comment. We have now extended our study period to cover the entire year 2021, following the implementation of travel restrictions based on color-coded zones and the widespread roll-out of the COVID-19 vaccination campaign in Belgium.

However, weekly travel-related data at the provincial level were only available up to early April 2022. For this reason, we chose to limit our analysis to the year 2021.

Q 1.2 *Data: the authors describe only how incidences on a regional level were collected. How did they obtain data on provincial level? Additionally, if data is available on a provincial level (for vaccination), why would you need another source for the regional information? Can you not simply sum the provincial data to get the regional data? Finally, it is not clear at which level the PLF data was available, regional or provincial?*

Reply: Thank you for this remark. To give more clarity, we have adjusted the Data subsection to now read:

> Data on daily confirmed COVID-19 cases and hospitalizations at the provincial level, along with weekly cumulative vaccinations at the municipality level, were made publicly available by Sciensano, the Belgian institute for public health. These data are available from `https://epistat.sciensano.be/covid/`.
>
> In 2021, every incoming traveler from abroad using any means of transportation had to fill in a passenger locator form (PLF) prior or upon arrival in Belgium (`https://dofi.ibz.be/sites/default/files/2021-07/PLF%20EN.pdf`). During this period, travelers arriving from high-risk zones were required to undergo mandatory testing on both day 1 and day 7 post-arrival. Sciensano documented the volume of incoming travelers and their test results in weekly epidemiological reports (`https://covid-19.sciensano.be/nl/covid-19-epidemiologische-situatie`, available in Dutch, French, and German). We extracted weekly data on incoming international travelers and the day 1 post-arrival positivity rate at the provincial level from these reports.

Q 1.3 *Statistical methods: it is unclear at which level each of the variables IC14w, IH7w, vfullw and travel are determined. The regional level section seems to suggest that it was on the provincial level, as that section states that the data was aggregated to regions.*
Why did the authors choose for a linear regression model at the regional level and a mixed model for the provincial level? You could have used a mixed model as well for the regions.
I fail to see the added value of the large tables with parameter estimates. The conclusions from the models seem contradictory in Table 1, as by going from model 1a to 1b/c the effect of travel is going from a negative effect to a positive effect. Moreover, in model 1c, at least the quadratic term seems unnecessary. Some statements in the text on the models are inconsistent. The authors state that in the end of section linear regression analysis, that model 2c has the same best fit as model 1c for a lag of 3 weeks. However, earlier in the section it was stated that the best lag for model 1c was a lag of 2 weeks. In table 2 and 3 the authors indicate the values of the residual variance as a significant random effect, which it is obviously not. The model description of models (3) and (4) do not coincide with the headers in table 2 and 3, for the variances.

Reply: Thank you for these comments. To provide a better focus of our results, we have removed the regional linear regression analysis from our revised manuscript. Due to this removal and major changes in our linear mixed model analysis, the results are presented according to these changes. We invite you to review these changes in the Results section.

Q 1.4 *Discussion: Does the overall conclusion on vaccination and travel changes in the model with the variable region added to the model?*

Reply: We have removed the linear regression analysis, therefore, we did not write any discussion about this topic in the revised manuscript. In the linear mixed model analysis, we used province as a random effect. Since Brussels is also a capital region, we did not include region as an explanatory variable to avoid overspecification of the model.

Q 1.5 *Abstract: incoming travel and vacc is associated with COVID-19 incidences (=cases? Or also includes hospitalizations?)*
Abstract: I'm not sure how surveillance, which mean to me 'only observe', can have an impact on COVID-19 transmission. One has to act before there can be an effect on transmission, one should not only observe.
Abstract: I would omit the sentence 'notable, proper transformations,..'. These are technical details of fitting the models, that may only be relevant for a statistically oriented audience.

Reply: We appreciate these remarks. We have revised the abstract to now read:

> In the absence of definitive treatments or vaccines, the primary strategy to mitigate the COVID-19 pandemic relied on non-pharmaceutical interventions. By the end of 2020, COVID-19 vaccines had been developed and initiated for preventive purposes. To better understand the association between various mitigation measures and their impact on the pandemic, we analyzed the effect of vaccination coverage, international travel, traveler positivity rates, and the stringency of public health measures on the incidence of COVID-19 cases and hospitalizations at the provincial level in Belgium. The analysis revealed crucial effects of these factors on the dynamics of COVID-19 cases and hospitalizations. Notably, the results suggest potential interactions among these variables, indicating that their combined effects may differ from the sum of their individual contributions. These findings underscore the intricate interplay between public health interventions, population immunity, and mobility patterns in shaping the course of the COVID-19 pandemic.

Q 1.6 *Introduction: Many would disagree that COVID-19 persists as a major health challenges at current times.*
Introduction: 'The original cause,... ': Is there another cause then SARS-CoV-2 for COVID-19? As far as I'm aware there are several variants of concern, but they still remain SARS-CoV-2.
Introduction: Why do the authors list the variants of concern until November 2021? This date seems very arbitrary. Why not until today or why not until the end of the period studied by the authors.
Introduction: Please define what you mean with 'full vaccination'. Do you mean full initiation or primary vaccination? Or does it mean primary vaccination + all boosters until July 2021?
Introduction: suggestion to replace '18+ population' by 'adult (>18 years) population'

Introduction: 'To meet the increasing need... necessary to develop more vaccines' This sentence is in present tense and I do not think there is currently an increasing need for vaccines, nor necessity to develop more vaccines.
Introduction: 'With the current availability,... lockdowns in 2022'. Currently we are in 2024.
Introduction: 'Many countries developed travel restrictions,...' Can the authors place this statement in a time period? Perhaps put in perspective when strict travel bans have been present in the country under study (Belgium).
Introduction: In the sentence 'These results were based mainly,....' replace 'simulation' by 'simulated'
Introduction: The second to last phrase is grammatically incorrect.

Reply: Thank you for raising these points. We have revised this section to provide more clarity about the recent COVID-19 situation around the world and in Belgium. We also corrected the phrases. We kindly invite you to review the revisions in the updated manuscript.

Q 1.7 *Data: Communities are not used in the analyses of the authors and not important for the interpretation of the results of the analyses, so I suggest to omit the sentences on communities and the figure 1b.*
Data: it is unclear at this stage what the authors mean with 'predictors'. It is only on section 'regional level' that the authors explain that these are the vaccination coverage and travel rate.

Reply: Thank you for these comments. Following your suggestions, we removed the explanation and figure related to communities. We also clarified the predictors used in our study.

Q 1.8 *Statistical methods: Consider to change 'we calculated first the weekly data of 14-day...' to 'we calculated the weekly 14-day...'*
Regional level: delete 'then' in 'We then explored various multiple linear regression models...'
Regional level: 'After thorough data exploration...' Can you add in the appendix a visualization of this non-linear relationship and how the inverse makes it linear? Also, the word 'thorough' can be omitted.
Regional level: The last part in the sentence 'Additionally, we took into account the lag...' seems to lack a verb.
Regional level: Did the authors not explore the association between travel and COVID-19 incidence and hospitalization in the same amount as for vaccination, that they allow for 'potential' non-linear effects?
Linear regression analysis: 'checked normality assumption' Did the authors mean that they evaluated if the errors were normally distributed?
Linear regression analysis: Since model 1 and 2 are the same in essence, would it not be more convenient to show a single formula and state that it is the best model for both outcomes?

Reply: Thank you for your input. Since we decided, based on earlier remarks, to remove the regional analysis from the paper, this section has been deleted. We invite the reviewer to read the new analysis section.

Q 1.9 *Figure 3 and 5 legend: replace 'outcomes' by 'COVID-19 incidences (top row) and COVID-19 hospitalization (bottom row)'.*

Reply: Thank you for this remark. Due to changes in our analysis, we have removed these figures.

Q 1.10 *Linear mixed model analysis: the first sentence is grammatically incorrect.*
Why did the authors not allow for a squared travel term for the linear mixed models?

Reply: Thank you for these comments. We have revised the Results section and we invite you to evaluate the changes presented in the Statistical Methods subsection.
Since we included a longer period and extra predictors in our analysis, we did not use a squared travel term to avoid overfitting.

Q 1.11 *Discussion: first sentence: 'summer 2020', Did the authors not explore April-July 2021?*
Discussion: Ukraine: which year?

Reply: Thank you for raising these points. We have revised the first paragraph to become:

> In this study, we identified important effects of lagged vaccination coverage, the rate of incoming international travel, the positivity rate among travelers, and the stringency index on the dynamics of COVID-19 confirmed cases and hospitalizations. Furthermore, our results emphasize the potential interactions among these variables, suggesting that their combined impact may be different than just the sum of their individual contributions.

We also included the study period reported in Ukraine. The sentence now reads:

> ... Similarly, research from Ukraine reported a surge in COVID-19 cases during the summer of 2021, attributed to increased travel and tourism in the absence of travel restrictions [28]. ...

Reviewer 2

The article "Empirical association between COVID-19 confirmed cases, hospitalization, vaccination, and travel [...]" by Yessika Adelwin Natalia et al. presents a statistical analysis between the mentioned explanatory and to be explained variables. One should maybe add "international" or better "air" in the title in front of travel, as other travel is not considered. The authors present an interesting and detailed view on the empirical association and I think that the final findings should be published. However, the paper is sometimes difficult to read and understand, has several several important issues and needs a major revision. Let me start with most important aggregated points:

Reply: We are grateful for your time and effort in reviewing our manuscript. To give more

clarity, we have added the word "international" in the title since the travel definition in our study included any means of transportation. The following suggestions have been taken into account.

***Q* 2.1** *Please avoid statements without references (see list below).*
Please repair all inconsistencies and unclear statements (see list below).
Please introduce models more explicitly (see list below).
Please improve overall writing (see list below).

Reply: Thank you. We have addressed these comments based on the provided list. A detailed response can be found from Q 2.9 onwards.

***Q* 2.2** *How did you include the different effects of vaccination against simple transmission (or severe disease) and how does this relate to your found k? There are some recent reviews on the time after (full) vaccination – Bobrovitz et al. Protective effectiveness of previous SARS-CoV-2 infection and hybrid immunity against the omicron variant and severe disease: a systematic review and meta-regression. The Lancet Infectious Diseases. 2023;https://doi.org/10.1016/S1473-3099(22)00801-5. – Stein C et al. Past SARS-CoV-2 infection protection against re-infection: a systematic review and meta-analysis. The Lancet. 2023;401(10379):833–842. doi:10.1016/s0140-6736(22)02465-5 but maybe immunity building between first and second vaccination should be considered, too.*

Reply: Thank you for this remark. Following your suggestion, we have adjusted our analysis to include longer lags up to six months. This is also mentioned in the Statistical Methods section as follows:

> . . . Taking into account that vaccination may have a delayed effect as immunity requires time to develop [22], we also explored the effect of vaccination coverage at lag l week as a potential predictor. Given the duration of our study period and the potential for waning immunity [23,24], we used the lagged vaccination coverage up to six months prior to the current week. This variable is denoted as $\text{vfull}_{i-l,j}$ with $l = 0, 1, \ldots, 24$. On top of this, we also considered the non-linear effect of vaccination and a decrease in the marginal benefit at high vaccination coverage [25,26]; thus we included the inverse of the vaccination coverage at lag l week, denoted as $\frac{10}{\text{vfull}_{i-l,j}}$, in our model selection.

***Q* 2.3** *It would be interesting to compare the number of incoming travellers against the data used in Klamser et al. Inferring country-specific import risk of diseases from the world air transportation network. PLOS Computational Biology. 2024;20(1):e1011775. doi:10.1371/journal.pcbi.1011775.*

Reply: Thank you for your suggestion. We have checked the data used in this study and we believe it was sourced from `https://migration-demography-tools.jrc.ec.europa.eu/data-hub/index.html`. Unfortunately, it covers only data between 2011 and 2016 (the study itself used data from 2014) at the country level. Therefore, these data might not be comparable with our current analysis.

Q 2.4 *The most important point: While you state (or want to infer) that air travel is a driver of confirmed cases, the manuscript does mention testing only a single time. I think the most important effect, the testing strategy has been neglected in this study. Confirmed cases are driven by tests and isolated air travelers do not spread (at least mostly). If the testing strategy (divided by "overall" and "at airports") was the same over all regions and all weeks of the study period, OK, but then please state it. If not, please mention this limitation and provide explanations where and how large this might have influenced your results.*

Reply: Thank you for raising this point. During our study period, travelers arriving from high-risk zones were required to undergo mandatory testing on both day 1 and day 7 post-arrival. This testing strategy remained stable in the whole country. We have included this explanation in the Data section to read:

> During this period, travelers arriving from high-risk zones were required to undergo mandatory testing on both day 1 and day 7 post-arrival.

Q 2.5 *Related to testing and travel: In Kuhn et al. Regional opening strategies with commuter testing and containment of new SARS-CoV-2 variants in Germany. BMC Infectious Diseases. 2022;22(1):333. doi:10.1186/s12879-022-07302-9 it was shown that high-incidence regions do not necessarily lead to larger incidences in surrounding regions if testing is sufficiently frequent. While this might be intuitive, this fact (and/or the quantification) should also be taken into consideration.*

Reply: Thank you for this suggestion. We included this topic in the Discussion section, as follows:

> The evidence collectively suggests that travel volume, particularly during peak periods like summer months, serves as a critical driver of COVID-19 transmission, especially when travelers originate from regions with high incidence rates [30]. However, such risks can be mitigated through targeted interventions, such as frequent testing of travelers and commuters. This approach not only helps identify and isolate potential cases but also reduces the likelihood of widespread transmission, even in contexts where contact reduction policies are less stringent [31]. These findings underscore the importance of integrating mobility management strategies with public health measures to curtail the spread of COVID-19 while balancing the socio-economic demands from the population.

Q 2.6 *Please provide an explanation why it is not a limitation that you consider air travel (mainly/solely) to regions/provinces with airports? What if Infected-Person A flies into Brussels Airport on Monday night but then actually moves to some province that same night?*

Reply: We appreciate this comment. In the passenger locator form, the travelers are asked to state their final destination in Belgium. This location is then used to group the travelers in the weekly report. We have added this information to the Statistical method section as follows:

> ... The weekly incoming travel rate (travel_{ij}) calculated as the number of incoming travelers (regardless of zone and mandate to get tested) to province j at week i per 100 inhabitants in that province (also denoted as percentage).

Q 2.7 *There is no explanation why you used different models for regional and provincial levels.*

Reply: Thank you for your input. We have removed the regional analysis from our revised manuscript to give a better focus on our results. We invite you to read the Statistical Methods subsection for more details regarding the provincial analysis.

Q 2.8 *Although PLOS asks authors to submit figures outside the manuscript upon acceptance, the review process would be facilitated if images appeared in the (initial) submission pdf where the caption is.*

Reply: Thank you for this suggestion. We have now included these figures in our revised manuscript.

Q 2.9 *Intro: "To meet the increasing need of vaccination around the world, it is necessary to develop more vaccines." → This is far too vague. Please highlight the problem more precisely and give some quantification with references.*
Intro: "The WHO reported that around 10 types of COVID-19 vaccine have been approved" → In which time frame?
p. 2, l. 30: depend[ED]
p. 2, l. 27-29: If you write it like this, the/some of the "few countries" that implemented lockdown should be named. And were this actually "few" if we look outside Europe?
p. 2, l. 40: It must have been the "CSF travel rush from January 26 to March 9, 2021" (as original source indicates) NOT 2020.

Reply: We are grateful for these remarks. We have revised this section to provide more clarity about the recent COVID-19 situation around the world and in Belgium. We also corrected the phrases mentioned above. We invite you to review these changes in the Introduction section.

Q 2.10 *What is the reason to consider different time frames for the incidences on cases and hospitalizations?*

Reply: Thank you for this comment. In the revised manuscript we used only 7-day incidences of confirmed cases and hospitalizations.

Q 2.11 *p. 3: It seems like the data you cite from https://covid-vaccinatie.be/en is no longer available. Can this data be provided or obtained on other ways?*

Reply: Thank you for raising this point. Due to the removal of the regional analysis, we did not include these data in our revised manuscript.

Q 2.12 *p. 3, l. 71-72: Please provide dates for the weeks X, Y, Z in parentheses. While some institutions tend to calculate in weeks, it is generally difficult to exactly grasp which*

date this really refers to.
p. 3, l. 73: Use a comma. It was probably the third wave at all, not the third wave of Alpha: "wave, predominantly driven by the Alpha variant."
p. 3, l. 74-76: Please rephrase difficult and long sentence.
p. 3, l. 79: "During this period" → You mentioned several before. Which one?
p. 3, l. 85: Why did you "calculate[d ...] the weekly data" of/for 14-day incidence of confirmed cases and 7-day hospitalizations? Do you mean "from the weekly data, you ...?
p. 3, l. 85-...: Rephrase the whole sentence which a) spans over six lines and b) uses "first" but no "second" or "then".
p. 3, l. 87: Do you mean vaccination rate (in the sense of the vaccination pace) or ratio ?
p. 3, l. 87: Written in 2024, it should be stated what "full" vaccination is and why it is/was named like this. Think of either explaining or renaming it. In 2021-2022, this might have been clear but today it is more than unclear.
p. 3, l. 95/96: "with incidence of COVID-19 confirmed cases or hospitalization as dependent variable" → You introduced abbreviations to make clear that the first a 14 days and the second 7 days. Use these abbreviations here.
p. 3, l. 97: "weekly 14-day incidence" is unclear. Rephrase or define it (or use abbreviations if this is the case).
p. 3, l. 104-...: Has the delayed approach been guided / informed (or validated afterwards) by the observed time (in public health / vaccine studies) for the vaccine to take "full effect" against any transmission?
p. 3, l. 107: Why did you include a quadratic travel term? It seems like you are mixing the results of a model selection process (if you did so? Did you come to this models by iteratively considering AIC/BIC and removing other quadratic terms etc.?)

Reply: We appreciate these comments. We have thoroughly adjusted our analysis to include only provincial level. In the revised analysis, we used AIC to select the best model. We accommodated the points raised and we invite you to evaluate the changes presented in the Statistical Methods subsection.

Q 2.13 *p. 4, l. 132: Rephrase or put "under investigation" at the end: Linear regression models of 14-day confirmed cases and 7-day hospitalizations under investigation are summarized in Table 1.*
p. 4, l. 133: What does "It should be noted that we checked the normality assumption for each model and we found no violation of this assumption." mean? Did you do a test (with which level) and obtained a p-value?
p. 4, l. 136...: Names like "Model 1a" should be capitalized through the text.

Reply: Thank you for these remarks. Since we decided, based on an earlier remark, to remove the regional analysis from the paper, this part has been deleted. We kindly ask you to assess the modifications in the revised version of the manuscript.

Q 2.14 *p. 5 and 6: As the regression and the description of the regional/province state is on the same level (subsection), it might be confusing/is not clear that the first regression is only done for one spatial resolution while the second model only applies to provinces. Please use subsubstructure or explain.*

p. 5, Table 1: Please define abbreviations such as for the standard error. Here, use the caption. The caption should also state more explicitly what "Travel", "Vaccination" and others mean in formulas. Use the caption.
p. 5, Table 1: x should not be used as multiplicator. Use ∗, × or directly the fraction of A/B instead of multiplying the denominator.
While it gets clear after some reading, it is difficult to grasp quickly what the 1 in model 1a, 1b, 1c stands for. I could make more sense to introduce a generic model in Eq. (1) for Model 1(a,b,c) with a variable term that you then name (and explicitly write down!) in another formula for a, b, and c, respectively.
p. 5, l. 145-147: If I am not mistaken, this only holds for vaccination lags of lower 50 days (top right image). Can you explain this?
p. 5, l. 155 (and before): How can the findings of $k = 2$ and $k = 3$ can be explained in the light of the different aggregation intervals, the different times before the vaccine might take effect against any transmission or severe cases and the time between reporting mild cases and hospitalization?

Reply: Thank you for raising these points. Based on earlier remarks, we have removed the regional linear regression analysis from our revised manuscript. Due to this removal and major changes in our linear mixed model analysis, the structure of the Result section has been considerably changed. We invite your attention to the updates incorporated in this section.

Q 2.15 *p. 6, l. 176: "Taking into account the results the regional level, we first fitted a random-intercepts model..." rightarrow This is rather vague. While you described the situation on the regional level, how does it differ from the province level?*
p. 6, l. 176: It is unclear/not defined what the "random-intercepts model" in "we first fitted a random-intercepts model" is.
p. 6, l. 179/180: "thereby properly taking the correlation within provincial sequences into account" → Does this mean that these correlations are not taken into account for the confirmed cases?
p. 6: Also here, all models are only defined implicitly or in text. Please provide formulas (maybe with a generic super-formula) and then substituting the particular variable by x, y, z for models a, b, c.
p. 6, l. 188/189: "However, the models incorporating random-intercepts-and-slopes exhibited only small improvements in AIC values" → I think this is even wrong! If I am not mistaken model this only applies for combination a/c but b/d show even slightly worsened outcomes.
p. 7, Table 2: See comments to Table 1.
p. 7, l. 184/185: "Considering low incidences and hospitalizations in some provinces, we modeled the logarithmic transformation of IC14pw and IH7pw" → Isn't this contradictory to the introductory sentences of the section ("fitted a random-intercepts model for the 14-day confirmed cases IC14pw")? Or are all results with transformation?

Reply: We appreciate your remarks. We have revised this section to clarify the final model notations. We welcome your feedback on the updates made in this revised version of the manuscript.

Q 2.16 *p. 8, l. 208: "during summer 2020" → I thought April to July?*
p. 8, l. 212–215: Deaths are mentioned here for the first time. I do not see a link to your paper. On the other hand, this general fact should be provable with more than just three US jurisdictions.
p. 8, l. 215–216: I do not see why higher vaccination rates would prevent breakthrough infections. At least, if I suppose correctly that rate is to be understood as a ratio. If you vaccinate faster, i.e., more often, I could understand but the simple fact that 10% more people are vaccinated would not prevent breakthrough infections. It would only/may prevent infections.
p. 8, l. 217–218: This misses proof.
p. 9, l. 225: "It is clear that travel volume in the summer months plays an important role in COVID-19 transmission." → please avoid these statements without proof.
p. 9, l. 226–228: I do not see why an imported case from a high-incidence region would make more impact than an imported case from a low-incidence region. It is one new case each.
p. 9, l. 233: "in a given week" → just in one week?
p. 9, l. 243–245: I do not get that sentence. Why is this particularly useful in a smaller area?
p. 9, l. 266/267: "Second, the Belgian stringency index has been constant over our study period." → In line 248 you stated the contrary!
p. 9, l. 267/268: "Hence, stringency cannot be taken up in the model and the results might 267" → I do not get that. not automatically hold when the stringency changes drastically.

Reply: Thank you for raising these points. To avoid misinterpretation, we used the term 'vaccination coverage' instead of 'vaccination rate' and rephrased the sentences. We have thoroughly revised the Discussion section based on the current methods and results. We kindly invite you to evaluate the changes presented in this section.

Q 2.17 *p. 10: The supporting information (data) should obtain a much better / more detailed description (with potential references to the article and its equations itself).*

Reply: Thank you for this comment. We have added more details in the description of the supporting files.
