# Supplementary material for: Empirical analysis of COVID-19 confirmed cases, hospitalizations, vaccination, and international travel across Belgian provinces in 2021
Source: PLoS One. 2025 May 23;20(5):e0322017. doi: 10.1371/journal.pone.0322017 (PMC12101632; doi:10.1371/journal.pone.0322017)
Supplement: Attachment — Submitted filename: review_2.pdf [file pone.0322017.s007.pdf]

Empirical association between COVID-19 confirmed cases, hospitalization, vaccination, and travel [...]

I highly appreciate the changes made by the authors, addressing all previously mentioned items. In the revised version, the authors have substantially improved the work and its presentation. I only have one larger and four minor items that I'd encourage the authors to address to further improve the presentation of the findings.

While the authors already include a lengthy discussion, I find that the evaluation and conclusion of findings is rather short and the authors miss to point to their most interesting findings. It is rather intuitive that confirmed cases variables improve hospitalization explanation but many other things pass uncommented. It is, e.g., interesting to see that both models find a similar lag for vaccination coverage to be considered. It is valuable that the authors provide the data and that every reader could look into the results, it would be valuable anyway to mention significance more prominent and rank and classify the different influence levels of vaccination, stringency, travel etc. This eventually, should also find its way to the conclusion.

Minor items/typos:

1. "To avoid confusion, the incidence of confirmed cases is denoted as $\log(IC7)_{ij}$": I suppose you mean "the logarithm of confirmed cases" or something similar?
2. "We observed a notable increase in the rate of incoming international travelers across all provinces in week 21": I suppose "from 21 onwards" was meant?
3. "March 29– -April 4": remove double hyphen
4. Fig. 2F: In your discussion you raise the point that the stringency index was only available on country level. It would be valuable to mention before the analysis (and potentially, in the discussion) if this was only of minor importance as NPIs for Belgium were mostly identical or if these differed a lot during different time frames.
