# Supplementary material for: Empirical analysis of COVID-19 confirmed cases, hospitalizations, vaccination, and international travel across Belgian provinces in 2021
Source: PLoS One. 2025 May 23;20(5):e0322017. doi: 10.1371/journal.pone.0322017 (PMC12101632; doi:10.1371/journal.pone.0322017)
Supplement: Attachment — Submitted filename: Response_to_reviewers_auresp_2.pdf [file pone.0322017.s008.pdf]

Empirical analysis of COVID-19 confirmed cases, hospitalizations, vaccination, and international travel across Belgian provinces in 2021

Yessika Adelwin Natalia[1], Geert Molenberghs[1,2], Thomas Neyens[1,2], Niel Hens[1,3], and Christel Faes[1]

[1] *I-BioStat, Data Science Institute, Hasselt University, Hasselt, Belgium*

[2] *I-Biostat, Leuven Biostatistics and Statistical Bioinformatics Centre, KU Leuven, Leuven, Belgium*

[3] *Centre for Health Economics Research and Modelling of Infectious Diseases (CHERMID), Vaccine and Infectious Disease Institute (VAXINFECTIO), University of Antwerp, Antwerp, Belgium*

Response to reviewers

Reviewer 2

I highly appreciate the changes made by the authors, addressing all previously mentioned items. In the revised version, the authors have substantially improved the work and its presentation. I only have one larger and four minor items that I'd encourage the authors to address to further improve the presentation of the findings.

Reply: Thank you very much for your appreciation. The following comments have been taken into account.

Q 1.1 *While the authors already include a lengthy discussion, I find that the evaluation and conclusion of findings is rather short and the authors miss to point to their most interesting findings. It is rather intuitive that confirmed cases variables improve hospitalization explanation but many other things pass uncommented. It is, e.g., interesting to see that both models find a similar lag for vaccination coverage to be considered. It is valuable that the authors provide the data and that every reader could look into the results, it would be valuable anyway to mention significance more prominent and rank and classify the different influence levels of vaccination, stringency, travel etc. This eventually, should also find its way to the conclusion.*

Reply: We are grateful for this comment. We provided the data used in our study as a supplementary material file (S1 File) and the full summary of each model is presented in the S2 File. In this way, the reader can reproduce our results.

While ranking the effects of individual explanatory variables is insightful, it becomes challenging in the presence of interaction terms, as the effect of one variable depends on the level of another. Given that our models incorporate up to three two-way interactions, isolating and interpreting their individual contributions would be very complex. Therefore, we have chosen a more general approach to presenting our results to ensure clarity and accuracy. We have revised the conclusion section to now read:

> Our findings highlight the complex interplay between public health interventions, population immunity, and mobility patterns in shaping the COVID-19 pandemic. We identified different interaction patterns among the covariates that influence the incidence of COVID-19 confirmed cases and hospitalizations. The best-fitting model for estimating confirmed cases (AIC = 965.658) revealed significant interactions between lagged vaccination coverage and the stringency index, as well as between incoming travel rates and positivity rates. When modeling COVID-19 hospitalizations, a significant interaction was observed between the incoming travel rate and the stringency index. Model performance improved substantially upon incorporating the incidence of confirmed cases as a covariate (AIC = 1,061.516 vs. AIC = 432.708), while highlighting key interactions between confirmed cases and traveler positivity rates, as well as between lagged vaccination coverage and incoming travel rates. For most optimal models, we identified a similar lag l of approximately 14-15 weeks. However, this lag duration should be interpreted with caution, as each model includes a different set of interaction terms. These insights contribute to a deeper understanding of the factors driving COVID-19 transmission and healthcare burden, offering valuable guidance for optimizing policy responses in future epidemic scenarios.

Q 1.2 *"To avoid confusion, the incidence of confirmed cases is denoted as* $\log(IC7)ij$*": I suppose you mean "the logarithm of confirmed cases" or something similar?*

Reply: Thank you for this remark. We have adjusted this sentence to now read:

> To avoid confusion, the logarithm of confirmed cases is denoted as $\log(\mathrm{IC7})_{ij}$ when used as an explanatory variable in the model.

Q 1.3 *"We observed a notable increase in the rate of incoming international travelers across all provinces in week 21": I suppose "from 21 onwards" was meant?*

Reply: Thank you for your suggestion, we have changed this sentence to now read:

> We observed a notable increase in the rate of incoming international travelers across all provinces from week 21 (May 24–30) onwards as illustrated in Fig 2D.

Q 1.4 *"March 29– -April 4": remove double hyphen.*

Reply: Thank you for bringing this to our attention, we have corrected the hyphen.

***Q* 1.5** *Fig. 2F: In your discussion you raise the point that the stringency index was only available on country level. It would be valuable to mention before the analysis (and potentially, in the discussion) if this was only of minor importance as NPIs for Belgium were mostly identical or if these differed a lot during different time frames.*

Reply: Thank you for raising this point. We have included more insight about the stringency index in the Data subsection and Discussion (paragraph 5 and 10) as follows:

> ... A higher stringency index indicates more stringent policies at the country level during a given period [25].
>
> ... At the beginning of 2021, the stringency index remained relatively high in response to the resurgence of infections during the fall of 2020 [40]. The restrictions were progressively eased starting from mid-April 2021 as part of a phased relaxation strategy (Fig 2F) following the nationwide vaccination rollout. The observed decline in stringency measures aligns with broader trends reported in European countries, where increased vaccination coverage facilitated policy shifts towards more lenient restrictions [41]. The strong association between vaccination coverage and the stringency index was further supported by our modeling results, which identified a significant interaction between these two variables (Eq 2 and 3, see also S2 File, Tables S2 and S3). ...
>
> It should be noted that the stringency index data are only available at the country level, therefore, they do not capture variations in policy stringency at lower administrative levels. ...

Reviewer 3

After reviewing it, I think the paper is very qualitative. In addition, given the fact that this is a revised version, the authors previously improved the overall level of their manuscript, this is why I will just give some minor comments in order to help to optimize the quality, if you don't mind here are some comments below :

Reply: We thank the reviewer for their time and effort in reviewing our revised manuscript and for the nice appraisal of our work. We have addressed the following comments and modified the paper accordingly.

***Q* 2.1** *In the abstract section : "The analysis revealed crucial effects of these factors on the dynamics of COVID-19 cases and hospitalizations. Notably, the results suggest potential interactions among these variables, indicating that their combined effects may differ from the sum of their individual contributions." I think you have to support your statements by adding some results (numbers).*

Reply: Thank you for raising this point. Considering the complexity of our models that incorporate up to three two-way interactions, including some numbers (e.g., coefficients,

standard error, or p-value) would be difficult, on top of the word count limit. We have revised the abstract by including extra details to give more clarity and accuracy, to now read:

> In the absence of definitive treatments or vaccines, the primary strategy to mitigate the COVID-19 pandemic relied on non-pharmaceutical interventions. By the end of 2020, COVID-19 vaccines had been developed and initiated for preventive purposes. To better understand the association between various mitigation measures and their impact on the pandemic, we analyzed the effect of vaccination coverage, international travel, traveler positivity rates, and the stringency of public health measures on the incidence of COVID-19 cases and hospitalizations at the provincial level in Belgium. We identified several important interactions among the covariates that influence the incidence of COVID-19 confirmed cases. Specifically, the best-fitting model (AIC = 965.658) revealed significant interactions between lagged vaccination coverage and the stringency index, as well as between incoming travel rates and positivity rates. Additionally, when modeling COVID-19 hospitalizations, a significant interaction was observed between the incoming travel rate and the stringency index. Model performance improved substantially when incorporating the incidence of confirmed cases as a covariate (AIC = 1061.516 vs. AIC = 432.708), while highlighting key interactions between confirmed cases and traveler positivity rates, as well as between lagged vaccination coverage and incoming travel rates. These findings underscore the intricate interplay between public health interventions, population immunity, and mobility patterns in shaping the course of the COVID-19 pandemic.

Q 2.2 *Lines 38-45, I appreciated the justification of your study. Nevertheless, I think you have to finish your abstract by clearly specifying the aim of your study, It may help the readers to better understand what you've done and why.*

Reply: We are grateful for this comment. We have revised the last paragraph of the Introduction section as follows:

> ...However, to our knowledge, no study has assessed the combined effect of COVID-19 vaccination and incoming international travel on confirmed cases and hospitalizations within a single analytical framework. Furthermore, it is crucial to account for additional factors that may influence disease dynamics, such as the positivity rate among incoming travelers and the public health mitigation measures implemented at different time points. To better understand the association between these factors and their impact on the COVID-19 pandemic, we conducted an analysis using publicly available data from Belgium. We focused our analysis on the year 2021, following the implementation of travel restrictions based on color-coded zones and the widespread roll-out of the COVID-19 vaccination campaign. By incorporating data on the positivity rate among incoming travelers and the mitigation measures in place during this time, we aim to provide a more comprehensive assessment of how international travel and vaccination jointly influenced COVID-19 transmission and healthcare burden.

Q 2.3 *Fig 1. Normally the scale should be put in the lower part of the map . In addition, please add the North direction and the source (eg. Authors) if you have drawn it.*

Reply: Thank you for this remark. We have adjusted Fig 1 to include the scale and north map. We referred the source of this map in the caption to now read:

> Border of provinces in Belgium. Regional division is marked with black lines. The map is adapted from [26] using R 4.4.1.

Q 2.4 *I suggest dividing your materials & methods section into 3 subsections: 1. Study Area 2. Data 3. Statistical Methods. You can include a revised version of your map and adding some information about Belgium in general.*

Reply: Thank you for your suggestion. We have divided the Materials and Methods section into three subsections and added general population information. We invite you to review these changes in the revised manuscript.

Q 2.5 *Isn't there more recent data than 2021? I think we should try to justify this by explaining this in the manuscript ?*

Reply: We appreciate this comment. We have included more explanation to justify our study period as follows:

> Until May 23, 2022, all incoming travelers to Belgium, regardless of their mode of transportation, were required to complete a Passenger Locator Form (PLF) either prior to or upon arrival [22]. During this period, travelers arriving from high-risk zones were required to undergo mandatory testing on both day 1 and day 7 post-arrival. Sciensano documented the volume of incoming travelers and their test results in weekly epidemiological reports (available in Dutch, French, and German) [23]. Detailed weekly data on international arrivals and the day 1 post-arrival positivity rate at the provincial level are available up until early April 2022. To maintain consistency in our data and analysis, we restricted our study period to include only data from the year 2021.

Q 2.6 *Line 171, you mean AIC= 432.708 ? If yes, please specify.*

Reply: This is indeed what we want to present. To clarify, we have changed this sentence into:

> Among these, the model achieving the lowest AIC value (432.708) is given by:

Q 2.7 *I think there is a lack of references in the discussion section, I recommend to discuss the findings of the following papers, or to read them (if not the case) in addition of other manuscripts, here are only examples: Doi: 10.1038/s41598-022-05498-z / Doi: 10.1016/j.ijbiomac.2022.01.118 / Doi: 10.3390/vaccines10111781 / Doi: 10.1080/01559982.2022.2045418*

Reply: We appreciate this input. Following the reviewer's suggestion, we have added the first two articles in the second paragraph of the Discussion section, as follows:

> Vaccination played a crucial role in reducing the incidence of COVID-19 cases and hospitalizations across various populations and geographic regions [32,33]. Consistent with these findings, our study demonstrates a significant association between vaccination coverage and COVID-19 incidences, as indicated by the negative coefficient of lagged vaccination coverage and/or its interaction term, while holding the other variables constant (S2 File, Tables S2–S4).

Further, we have expanded some parts of our discussion and added extra references. We invite you to review these changes in the revised manuscript.

Q 2.8 *Same comment as the abstract section, please consider improving your discussion by emphasizing some quantitative results to support some statements.*

Reply: Thank you for raising this point. As mentioned in Q 2.1, it would be difficult to include quantitative results related to the fitted models given the complexity of the interaction terms. We tried, however, to give better explanation and comparison with other studies in the Discussion. We invite you to review these changes in the revised manuscript.

Q 2.9 *Line 258-291, please avoid using first, second,... etc while describing your limitations.*

Reply: Thank you for this remarks. We have removed the word 'first', 'second', and 'third' and replaced it with 'On top of this' and 'Further'.

Q 2.10 *Conclusion sections looks a little weak, please try to extend it a bit.*

Reply: Following your suggestion, we have revised the conclusion to now read:

> Our findings highlight the complex interplay between public health interventions, population immunity, and mobility patterns in shaping the COVID-19 pandemic. We identified different interaction patterns among the covariates that influence the incidence of COVID-19 confirmed cases and hospitalizations. The best-fitting model for estimating confirmed cases (AIC = 965.658) revealed significant interactions between lagged vaccination coverage and the stringency index, as well as between incoming travel rates and positivity rates. When modeling COVID-19 hospitalizations, a significant interaction was observed between the incoming travel rate and the stringency index. Model performance improved substantially upon incorporating the incidence of confirmed cases as a covariate (AIC = 1,061.516 vs. AIC = 432.708), while highlighting key interactions between confirmed cases and traveler positivity rates, as well as between lagged vaccination coverage and incoming travel rates. For most optimal models, we identified a similar lag l of approximately 14-15 weeks. However, this lag duration should be interpreted with caution, as each model includes a different set of interaction terms. These insights contribute to a deeper understanding of the factors driving COVID-19 transmission and healthcare burden, offering valuable guidance for optimizing policy responses in future epidemic scenarios.

Q 2.11 *The references are well chosen and the majority are recent, it was a good job. Again, I just suggest to increase their number in order to better support your work.*

Reply: Thank you very much for your input. We have expanded some parts of our discussion and added extra references. We invite you to review these changes in the revised manuscript.
